# Effects of aquatic and land high intensity interval training on hemodynamics and vascular function of middle-aged men

**DOI:** 10.3389/fphys.2024.1411277

**Published:** 2024-07-12

**Authors:** Chenxi Xin, Jiahao Fu, Zhihui Zhou, Yujiao Zhou, Hui He

**Affiliations:** ^1^ Physical Education Department, Shanghai University of Finance and Economics, Shanghai, China; ^2^ China Institute of Sports and Health, Beijing Sport University, Beijing, China; ^3^ Physical Education Department, Zhejiang Guangsha Vocational and Technical University of Construction, Zhejiang, China; ^4^ Physical Education Department, Anhui Science and Technology University, Anhui, China; ^5^ Physical Education Department, Beijing University of Aeronautics and Astronautics, Beijing, China

**Keywords:** aquatic HIIT, hemodynamics, middle-aged men, vascular function, HIIT

## Abstract

**Objective:** To investigate the effects of 8-week aquatic and land high intensity interval training (HIIT) on hemodynamics and vascular function in middle-aged men.

**Methods:** Thirty middle-aged men with low physical activity were selected and divided into 15 men (52.43 ± 4.11) in aquatic group and 15 men (52.74 ± 5.62) in land group by random number table. They performed HIIT exercise in aquatic and land 3 times a week for 8 weeks. Pre-test, inter-test and post-test respectively measure hemodynamics and blood vessel function.

**Results:** (1) Body composition: After 8 weeks of exercise, weight, body mass index (BMI) and body fat rate (BF) were lower than before exercise (aquatic group: *p* < 0.01, land group: *p* < 0.05). The improvement of BF in the aquatic group was better than that in the land group (*p* < 0.05); (2) Cardiac function: After 8 weeks of exercise, stroke volume (SV), left ventricular end-diastolic volume (EDV), cardiac output (CO), and left ventricular fractional shortening (FS), were higher than before exercise (aquatic group: *p* < 0.01, land group: *p* < 0.05), heart rate (HR) and left ventricular end-systolic volume (ESV) were lower than before exercise (aquatic group: *p* < 0.01, land group: *p* < 0.05). The improvement of SV, HR, EDV, ESV, CO and FS in the aquatic group was better than that in the land group (*p* < 0.05); (3) Hemodynamics: After 8 weeks of exercise, systolic blood pressure (SBP), diastolic blood pressure (DBP) were lower than before exercise (aquatic group: *p* < 0.01, land group: *p* < 0.05), wall shear stress (WSS) and peak systolic velocity (PSV) were higher than before exercise (aquatic group: *p* < 0.01, land group: *p* < 0.05). The improvement of SBP, WSS and PSV in the aquatic group was better than that in the land group (*p* < 0.05); (4) Vascular function: basal diameter and brachial artery flow-mediated dilatation (FMD) level in aquatic group and land group was higher than before exercise, pulse wave velocity (PWV) level was lower than before exercise (aquatic and land group: *p* < 0.05). The improvement of FMD in the aquatic group was better than that in the land group.

**Conclusion:** The body composition, hemodynamics and vascular function of middle-aged men were improved by 8-week aquatic and land HIIT. Aquatic HIIT has better effect on body fat rate, hemodynamics and vascular endothelial function in middle-aged men due to the effect of aquatic pressure and temperature.

## 1 Introduction

Cardiovascular disease is the main cause of global medical burden and mortality (Roth et al., 2020). How to prevent the occurrence of cardiovascular disease and explore its pathogenesis are the current research hotspots. According to the latest study in 2022, the prevalence and mortality of cardiovascular disease in men are higher than those in women due to differences in living habits and physical activity levels (Li et al., 2022). In the middle-aged population, the decline of vascular endothelial function in men is faster than that in women, and the prevalence of cardiovascular disease in middle-aged men is also higher than that in middle-aged women (Celermajer et al., 1994). In addition, compared to the elderly population, the heart of the middle-aged population exhibits considerable plasticity and is capable of responding to an appropriate exercise intervention in sufficient doses to improve myocardial atrophy and stiffness (Howden et al., 2018). Therefore, it is particularly crucial for middle-aged men to proactively prevent and delay the onset of cardiovascular disease.

Studies have found that factors such as hemodynamics and vascular endothelial injury are closely related to the formation, progression and treatment of cardiovascular diseases. For example, Wall shear stress (WSS) and abnormal blood pressure can contribute to the decline of cardiovascular function, ultimately leading to heart failure, atherosclerosis, and other cardiovascular diseases (Souilhol et al., 2020). Vascular stiffness and functional changes are the main pathological basis of cardiovascular diseases (Witte et al., 2005). Early identification of arterial lesions is helpful for early diagnosis and prevention of complications (Li et al., 2022). Brachial artery flow-mediated dilatation (FMD) (Ras et al., 2013) and Pulse wave velocity (PWV) (Tomiyama et al., 2016) can measure vascular endothelial function and vascular stiffness to assess vascular functional status. It can also be used as an important reference index for the diagnosis and treatment of cardiovascular diseases (Celermajer et al., 1992). Therefore, the simultaneous management of hemodynamics and vascular function is an important means to prevent cardiovascular diseases.

It is well known that scientific and regular physical exercise can reduce the incidence of cardiovascular diseases (Beral and Million Women Study Collaborators, 2003). Exercise can improve cardiovascular function mainly by enhancing myocardial contractility and regulating hemodynamics, so as to improve cardiovascular dysfunction (Green et al., 2017). At present, due to the different factors such as exercise style and intensity, the research results are mixed. High Intensity Interval Training (HIIT) has a good effect on improving blood pressure and vascular endothelial function, and the improvement effect is similar to or better than that of moderate intensity aerobic exercise (He et al., 2022). Compared with land exercise, aquatic exercise has better improvement benefits on vascular function, blood pressure and other physiological aspects, and the improvement benefits of aquatic exercise are better than that of land exercise (Saowaluck et al., 2016). The body immersed in water will produce hydrostatic pressure gradient and increase the pressure on the surface of the body due to the particularity of water environment, thus increase the venous return, the blood volume of the heart and blood vessels (Murray, 1976). However, most of the studies on aquatic exercise focus on the acute effects on blood pressure and other indicators, and there are few studies on the adaptability of aquatic exercise to improve vascular function and the difference of improvement compared with land exercise. Therefore, it remains to be explored whether aquatic environment combined with HIIT exercise has better effects on hemodynamics and vascular function than land HIIT exercise. By comparing the effects of 8-week aquatic and land HIIT exercise on hemodynamics and vascular function of middle-aged men, this study investigated the effect of aquatic HIIT exercise on improving hemodynamics and vascular function, providing theoretical and practical basis for improving hemodynamics and vascular function.

## 2 Methods

### 2.1 Participants

A total of 30 middle-aged men with low physical activity were selected and divided into 15 in the aquatic HIIT group and 15 in the land HIIT group using a random number table (after excluding the attendance rate of less than 95%, the number of people who finally completed the experimental intervention was distributed as follows: 14 in the aquatic HIIT group and 13 in the land HIIT group); Participants were asked to maintain their original physical activity levels and dietary habits during the intervention period; All subjects signed informed consent before participating in the experiment. The research protocol was approved by the Beijing Sport University Ethics Committee (2022191H). The basic information of subjects is shown in Table 1. The subjects inclusion criteria were as follows: (1) middle-aged men aged 45–59 years (Ahmad et al., 2001) with a height of 165–170 cm; (2) No cardiovascular disease and other diseases that affect physical exercise, and no drugs such as lowering blood lipids and blood pressure have been taken recently; (3) no alcoholism, tobacco dependence; (4) In addition to this experiment, no longer participate in any other form and any organization of exercise training; (5) Middle-aged men who reported low levels of Physical Activity on the International Physical Activity Questionnaire (IPAQ).

**TABLE 1:** Basic information of subjects.

Group	N	Age (years)	Height (cm)	Weight (kg)	BMI (kg/m2)	IPAQ (MET-min/w)
Aquatic group	15	52.43 ± 4.11	167.39 ± 2.01	73.62 ± 5.53	25.23 ± 1.91	431 ± 73.23
Land group	15	52.74 ± 5.62	167.71 ± 2.12	72.82 ± 4.44	25.31 ± 1.52	425 ± 76.11

BMI, body mass index; IPAQ, international physical activity questionnaire. Values are mean ± SD.

### 2.2 Exercise intervention

HIIT exercise program was developed with reference to previous studies (Tang et al., 2022). And two groups of subjects were subjected to aquatic and land HIIT exercise intervention 3 times a week for 8 weeks. The aquatic group received exercise intervention in a swimming pool with a water depth of 1.2 m and a water temperature of 26°C–28°C (Haynes et al., 2021). The body temperature of the subjects’ rectus femoris and the water temperature in the swimming pool were recorded before and after the intervention, as shown in Table 2. The land group received exercise intervention on the professional track (without slope) of Beijing Sport University. Before the intervention, the heart rate reserve (HRR) of each subject was calculated according to the resting heart rate (RHR) and maximum heart rate (MHR = 220-age), and the target heart rate during exercise was determined (THR = RHR+ 80–85% MHR-RHR). The changes in exercise heart rate in each group are shown in Table 3. All subjects wore a heart rate monitor (Polar Team 2, Finland) to control their exercise intensity. Each training session is 40 min in total, 10 min of warm-up (35%-40%HRR), 18–20 min of training (30s fast running ×12 sets, 80–85%HRR, 1 min complete rest between groups), 10 min of relaxation. During the first week of intervention, subjects completed training at 75–80%HRR. The exercise intervention was carried out at 80–85%HRR intensity from the second week until the end of the last training session.

**TABLE 2:** Temperature records of aquatic group.

Variable	N	Before class	After class
Body temperature	15	36.57 ± 1.11	32.12 ± 1.15**
Water temperature	15	26.34 ± 0.62	26.56 ± 0.32

Values are mean ± SD.*/** indicates the comparison between pre-test and post-test (*p* < 0.05/0.01).

**TABLE 3:** Panel A Changes in Heart Rate during exercise. Panel B Effects of aquatic and land HIIT on body composition of middle-aged men.

Group	RHR (bpm)	MHR (bpm)	THR (bpm)	EHR (bpm)
Aquatic group	71.57 ± 3.56	167.49 ± 4.01	144–157	150.23 ± 4.13
Land group	71.61 ± 3.51	166.96 ± 5.29	143–158	149.82 ± 5.44

Variable	Aquatic group (*n* = 14)					Land group (*n* = 13)			
	Pre-test	Inter-test		Post-test	ES	Pre-test	Inter-test	Post-test	ES
Weight (kg)	73.31 ± 7.52	71.21 ± 7.41^*^		69.75 ± 7.51^**#^	0.47	72.21 ± 6.42	71.13 ± 6.11^*^	70.34 ± 6.21^*#^	0.29
BMI (kg/m^2^)	25.51 ± 2.11	24.12 ± 1.72^*^		23.13 ± 1.53^**#^	1.29	25.15 ± 1.54	24.32 ± 1.55^*^	23.32 ± 1.64^*#^	1.15
BF (%)	27.74 ± 3.72	26.92 ± 3.53^*^		25.11 ± 3.65^**#△^	0.71	27.43 ± 3.42	26.83 ± 3.33^*^	25.71 ± 3.44^*#^	0.50

RHR: resting heart rate; MHR: maximum heart rate THR: target heart rate; EHR: exercise heart rate.

BMI, body mass index; BF, body fat rate; ES, effect sizes. Values are mean ± SD. *p < 0.05, **p < 0.01, *versus* Pre-test within group; #*p* < 0.05 or ##*p* < 0.01, represents the comparison between Inter-test and Post-test; △*p* < 0.05, *versus* among groups.

### 2.3 Measurements

Hemodynamics and vascular function were tested before exercise intervention, at the end of the fourth and eighth week respectively. Requirements: Subjects were tested before, during and after the exercise intervention 24 h after the interval, and did not smoke, drink strong tea, coffee or alcohol 12 h before the test. All examinations were performed by the same physician on the same day, and the ambient temperature of the test was 24°C–26°C.

Temperature was measured using a hand-held temperature meter (HT3300, Italy). Height and weight were measured using a height meter and weight measuring instrument (SG-1001, China). Body mass index (BMI) was calculated from height and weight. Body composition was tested by Dual energy X-ray absorptiometry (LunarDPX Prodigy, United States) for each subject (Messina et al., 2020). The subjects were asked to fasting, avoid strenuous exercise, wear non-metallic clothing, and the test was carried out after the physiological characteristics were stabilized while lying still for 5 min. The data such as Body fat rate (BF) obtained by whole body scanning were recorded.

Cardiac function tests were performed using color Doppler ultrasound (GEvivid 7, United States) (Oglat et al., 2018). The subjects were asked to rest on the test bed for 10 min, and start the test while their heart rate was stable. The ultrasound probe was placed in the third to fourth rib on the left side of the sternum, with the direction pointing to the left shoulder. Analysis indicators: Left ventricular end-diastolic volume (EDV), Left ventricular end-systolic volume (ESV), Stroke volume (SV), Left ventricular fractional shortening (FS), Cardiac output (CO), Heart rate (HR).

Hemodynamic tests were performed using color Doppler ultrasound (GEvivid 7, United States) (Oglat et al., 2018). The subjects were asked to rest supine on the test bed for 10 min, and start the test while their heart rate was stable. The Peak systolic velocity (PSV), diameter waveform, pulsation index and resistance index of the common carotid artery were measured at the position 1–1.5 cm before the carotid sinus bifurcation. The blood flow velocity and diameter of the common carotid artery were measured into the formula. The calculation formula is as follows: WSS = 4ηV/D, where η is the blood viscosity, V is the blood flow velocity, D is the inner diameter of the blood vessel, and the blood viscosity η = 4 mPa s was used for the calculation.

Endothelium-dependent flow-mediated endothelial relaxation of the brachial artery was tested using a vascular endothelial function tester (UNEX EF, Japan) (Corretti et al., 2002). The subjects were asked to lie supine on the test bed in the resting state for 10 min, and the test was performed after the physiological characteristics of the body had stabilized. Participants were instructed to put their arms near to the body, whilst the blue compression band was tied to the position above the elbow joint, and two clips were clamped on the wrist of the subject’s left arm. The right arm was freely placed on the mat of the operating desk, and the red compression band was tied to the position above the wrist, with the palm fixed on the operating desk. The tightness between the band and arm through which two fingers could be fitted was considered eligible. Couplant was coated on the humerus about 3–5 cm above the elbow joint of subject’s right arm, and the Type-H probe was then located on the brachial artery. When a clear image emerged on the screen, the probe was fixed, and the resting Systolic blood pressure (SBP), Diastolic blood pressure (DBP), brachial vessel diameters with blood flow velocity were measured. At the end of the measurement, the subject was required to remain motionless, given a 50 mmHg boost based on quiet blood pressure, and performed 5 min of exercise. After 5 min, the pressure was automatically released, whilst the inner diameter and blood flow velocity within 2 min were automatically recorded, and the FMD was calculated by computer.

PWV was measured by Omron arteriosclerosis detector (BP-203RPEIII, Japan) (Takashima et al., 2014). The subjects were asked to rest supine on the test bed for 10 min, and the test was performed after while their heart rate was stable. During the measurement, the sensor was placed at the pulse points of the subject’s neck and femoral artery. The pulse waveform and time difference of the two points were recorded by the detector at the same time, and the PWV value was automatically calculated using the standard formula.

### 2.4 Statistics and analysis

SPSS19.0 statistical software was used to process the experimental data. The data of each group were in accordance with normal distribution by S-W test (Skewness: 0.71, kurtosis:2.12). There was no significant difference in baseline data between the two groups after independent sample *t*-test. Repeated measures analysis of variance was used to compare the intra-group data and inter-group changes of the two groups before, during and after the intervention. The between-group factors were group (aquatic group, land group), and the within-group factors were time (before exercise, 4 weeks of exercise, 8 weeks of exercise). When there was an interaction effect of time * group, the simple effect analysis was performed on each factor. Effect sizes (ES) were calculated by Cohen’s d. Statistical description was expressed as “Mean ± standard deviation” (Mean ± SD). *p* < 0.05 was accepted as the level of statistical singnificance.

## 3 Results

### 3.1 Effects of aquatic and land HIIT on body composition of middle-aged men

As shown in Table 3, after an 8-week exercise intervention, there was a time * group interaction effect on body weight (F = 4.97, *p* = 0.028), BMI (F = 4.11, *p* = 0.031), and BF (F = 4.57, *p* = 0.038) between the aquatic group and the land group (*p* < 0.05). The improvement of BF in the aquatic group was better than that in the land group (*p* < 0.05). After 4 weeks of exercise, the body weight, BMI and BF levels of the aquatic group and the land group were lower than those before exercise (*p* < 0.05). After 8 weeks of exercise, the body weight, BMI and BF of the aquatic group and the land group were significantly lower than those before exercise (aquatic group: *p* < 0.01, land group: *p* < 0.05). After 8 weeks of exercise, the body weight, BMI and BF levels of the aquatic and land group were significantly lower than those of the 4 weeks of exercise (*p* < 0.05).

### 3.2 Effects of aquatic and land HIIT on cardiac function of middle-aged men

As shown in Table 4, after an 8-week exercise intervention, there was a time * group interaction effect on SV (F = 4.22, *p* = 0.041), HR (F = 3.58, *p* = 0.036), EDV (F = 4.87, *p* = 0.031), ESV (F = 4.53, *p* = 0.035), CO (F = 4.23P = 0.041) and FS (F = 3.45, *p* = 0.025) between the aquatic group and the land group (*p* < 0.05). The improvement of SV, HR, EDV, ESV, CO and FS in the aquatic group was better than that in the land group (*p* < 0.05). After 4 weeks of exercise, SV, EDV, CO and FS levels in aquatic group were higher than before exercise (*p* < 0.05), HR and ESV levels were lower than before exercise (*p* < 0.05), HR level in land group was lower than before exercise (*p* < 0.05), SV, ESV and FS levels had no significant difference compared with before exercise (*p* > 0.05). After 8 weeks of exercise, SV, EDV, CO and FS in aquatic group and land group were higher than before exercise (aquatic group: *p* < 0.01, land group: *p* < 0.05), and HR and ESV levels were lower than before exercise (aquatic group: *p* < 0.01, land group: *p* < 0.05). After 8 weeks of exercise, SV, EDV, CO and FS levels in aquatic group and land group were higher than those in 4 weeks of exercise (*p* < 0.05), while HR and ESV levels were lower than those before exercise (*p* < 0.05).

**TABLE 4:** Effects of aquatic and land HIIT on cardiac function of middle-aged men.

Variable	Aquatic group (*n* = 14)					Land group (*n* = 13)							
	Pre-test	Inter-test	Post-test	ES		Pre-test		Inter-test		Post-test		ES	
SV (mL)	50.40 ± 4.64	55.13 ± 7.16^*^	59.39 ± 7.21^**#△^	1.48		50.70 ± 5.38		50.99 ± 5.78		54.30 ± 7.16^*#^		0.57	
HR (bpm)	71.57 ± 3.56	68.64 ± 3.42^*^	65.34 ± 3.38^**#△^	1.79		71.61 ± 3.51		69.26 ± 3.54^*^		68.96 ± 3.24^*#^		0.78	
EDV (mL)	117.52 ± 4.96	120.56 ± 5.48^*^	123.43 ± 5.28^**#△^	1.15		117.59 ± 4.64		118.52 ± 4.74^*^		119.62 ± 4.48^*#^		0.45	
ESV (mL)	67.12 ± 4.32	65.43 ± 4.36^*^	64.04 ± 4.35^**#△^	0.71		66.89 ± 4.62		67.13 ± 4.47		65.32 ± 4.54^*#^		0.34	
CO (L)	4.43 ± 0.82	5.29 ± 0.76^*^	5.84 ± 0.85^**#△^	1.68		4.49 ± 0.84		4.91 ± 0.67^*^		5.45 ± 0.78^*#^		1.18	
FS (%)	25.94 ± 3.62	28.44 ± 2.62^*^	30.94 ± 2.92^**#△^	1.52		25.89 ± 3.23		26.14 ± 3.12		28.53 ± 3.31^*#^		0.81	

EDV, ventricular end-diastolic volume; ESV, ventricular end-systolic volume; CO, cardiac output; FS, ventricular fractional shortening; ES, effect sizes. Values are mean ± SD. *p < 0.05, **p < 0.01, *versus* Pre-test within group; #*p* < 0.05 or ##*p* < 0.01, represents the comparison between Inter-test and Post-test; △*p* < 0.05, *versus* among groups.

### 3.3 Effects of aquatic and land HIIT on hemodynamics of middle-aged men

As shown in Table 5, after an 8-week exercise intervention, there was a time * group interaction effect on SBP (F = 5.23, *p* = 0.035), DBP (F = 4.37, *p* = 0.041), WSS (F = 3.23, *p* = 0.033) and PSV (F = 4.32, *p* = 0.029) between the aquatic group and the land group (*p* < 0.05). The improvement of SBP, WSS and PSV in the aquatic group was better than that in the land group (*p* < 0.05). After 4 weeks of exercise, the SBP and DBP in the aquatic group and the land group were lower than those before exercise (*p* < 0.05), and the WSS and PSV levels in the aquatic group were higher than those before exercise (*p* < 0.05), while the WSS and PSV levels in the land group were not significantly different from those before exercise (*p* > 0.05). After 8 weeks of exercise, SBP and DBP in the aquatic group and land group were lower than those before exercise (aquatic group: *p* < 0.01, land group: *p* < 0.05), WSS and PSV levels were significantly higher than those before exercise (aquatic group: *p* < 0.01, land group: *p* < 0.05). After 8 weeks of exercise, SPB and DBP in the aquatic group and land group were lower than those before exercise (*p* < 0.05), and WSS and PSV levels were significantly higher than those after 4 weeks of exercise (*p* < 0.05).

**TABLE 5:** Effects of aquatic and land HIIT on hemodynamics of middle-aged men.

Variable	Aquatic group (n = 14)				Land group (n = 13)			
	Pre-test	Inter-test	Post-test	ES	Pre-test	Inter-test	Post-test	ES
SBP(mmHg)	128.53 ± 8.13	125.56 ± 7.85^*^	123.74 ± 7.54^**#△^	0.61	128.82 ± 8.54	126.39 ± 8.78^*^	124.33 ± 8.63^*#^	0.52
DBP(mmHg)	77.91 ± 8.35	75.15 ± 8.97^*^	74.72 ± 7.61^**#^	0.40	78.3 ± 7.12	77.2 ± 6.41^*^	76.62 ± 6.51^*#^	0.25
PSV (cm/s)	67.82 ± 5.34	70.88 ± 5.95^*^	74.39 ± 5.76^**#△^	1.18	66.33 ± 5.71	66.77 ± 5.42	68.23 ± 5.43^*#^	0.34
WSS (dynes/cm^2^)	14.69 ± 2.33	15.42 ± 1.94^*^	17.41 ± 1.86^**#△^	1.29	14.65 ± 1.96	14.84 ± 1.76	15.31 ± 1.93^*#^	0.34

SBP, systolic blood pressure; DBP, diastolic blood pressure; PSV, peak systolic velocity; WSS, wall shear stress; ES, effect sizes. Values are mean ± SD. *p < 0.05, **p < 0.01, *versus* Pre-test within group; #*p* < 0.05 or ##*p* < 0.01, represents the comparison between Inter-test and Post-test; △*p* < 0.05, *versus* among groups.

### 3.4 Effects of aquatic and land HIIT on vascular function of middle-aged men

As shown in Table 6, after an 8-week exercise intervention, there was a time * group interaction effect on basal diameter (F = 3.23, *p* = 0.043), FMD (F = 4.23, *p* = 0.025) and PWV (F = 6.23, *p* = 0.045) between the aquatic group and the land group (*p* < 0.05). The improvement of FMD in the aquatic group was better than that in the land group (*p* < 0.05). After 4 weeks of exercise, FMD level in aquatic group was higher than before exercise (*p* < 0.05), FMD level in land group was not significantly different compared with before exercise (*p* > 0.05), basal diameter and PWV level in aquatic group and land group was not significantly different compared with before exercise (*p* > 0.05). After 8 weeks of exercise, basal diameter and FMD level in aquatic group and land group was higher than before exercise (aquatic group: *p* < 0.01, land group: *p* < 0.05), PWV level was lower than before exercise (*p* < 0.05). After 8 weeks of exercise, basal diameter and FMD level in aquatic group and land group was higher than that of exercise for 4 weeks (*p* < 0.05), and the PWV level was lower than that of exercise for 4 weeks (*p* < 0.05).

**TABLE 6:** Effects of aquatic and land HIIT on vascular function of middle-aged men.

Variable	Aquatic group (n = 14)				Land group (n = 13)			
	Pre-test	Inter-test	Post-test	ES	Pre-test	Inter-test	Post-test	ES
basal diameter (mm)	3.84 ± 0.62	3.79 ± 0.77	4.22 ± 0.72*#	0.56	3.78 ± 0.71	3.82 ± 0.78	4.10 ± 0.61*#	0.48
FMD (%)	8.14 ± 1.22	9.02 ± 1.24*	10.63 ± 1.42**#△	1.88	7.89 ± 1.28	7.96 ± 1.13	8.62 ± 1.22*#	0.58
PWV (cm/s)	1,405.73 ± 181.55	1,407.42 ± 143.11	1,351.64 ± 149.28*#	0.32	1,403.53 ± 152.13	1,403.93 ± 147.88	1,357.54 ± 146.68*#	0.30

FMD, flow-mediated dilatation; PWV, pulse wave velocity; ES, effect sizes. Values are mean ± SD. *p < 0.05, **p < 0.01, *versus* Pre-test within group; #*p* < 0.05 or ##*p* < 0.01, represents the comparison between Inter-test and Post-test; △*p* < 0.05, *versus* among groups.

## 4 Discussion

This study investigated the effects of aquatic and land HIIT on hemodynamics and vascular function of middle-aged men. The results showed that both aquatic HIIT and land HIIT 3 times a week for 8 weeks could improve body composition, hemodynamics and vascular function in middle-aged men, and aquatic HIIT improved BF, SV, HR, EDV, ESV, CO, SBP and FMD more significantly.

Exercise can significantly improve the body weight, BF and BMI of middle-aged sedentary men (Donges et al., 2013). This study found that both aquatic HIIT and land HIIT can significantly improve body weight, BF and BMI, which are the result of increased energy expenditure caused by long-term exercise. Due to the particularity of water environment, the body loses heat in water faster than in land movement, so that the energy consumed by the subjects in water movement is much higher than that on land (Pendergast et al., 2015). Therefore, aquatic HIIT can reduce the body fat percentage of middle-aged men more effectively under the same intensity.

Studies have shown that regular exercise is beneficial to lower resting heart rate of subjects (Cornelissen et al., 2010) and increase EDV levels (Vogelsang et al., 2008). However, different exercise methods have different effects on middle-aged men. Resistance exercise can increase peripheral resistance or afterload, leading to cardiac hypertrophy (Ruiz et al., 2011). Aerobic exercise increases venous return and blood volume or preload, and promotes myocardial centrifugal hypertrophy (Heiskanen et al., 2016). HIIT exercise has stronger stimulation on cardiovascular function and greater adaptive changes in cardiovascular function during the period of high-intensity exercise. Although the exercise intensity in the interval period is at a lower level, the body’s metabolism is still at a higher level (Gibala et al., 2012). Therefore, HIIT can more effectively improve the cardiac function and increase the stroke volume and cardiac output (Ramos et al., 2015). This study found that aquatic HIIT reduced sympathetic nerve activity, stimulated subcutaneous capillaries, and increased venous blood return to the heart due to the influence of hydrostatic pressure and water temperature, thereby reducing heart rate and increasing output per wave and cardiac output (Reilly et al., 2003). Therefore, the effect of aquatic HIIT is better under the same conditions.

Through mesh meta-analysis, Chenxi Xin (Xin et al., 2022)et al. found that exercise was conducive to improve abnormal blood pressure, and water exercise was superior to traditional aerobic or resistance exercise in improving SBP indexes. The improvement mechanism of exercise on blood pressure of middle-aged men is mainly through accelerating blood circulation, enhancing the impact force and shear stress of blood on the inner wall of blood vessels, carrying away the inner wall deposits of blood vessels, reducing peripheral resistance, and thus improving the compliance of peripheral blood vessels (Ashor et al., 2014). It can also improve vascular endothelial function and promote vascular dilation by increasing the release of nitric oxide, thus effectively reduce blood pressure (Moncada and Higgs, 2006). This study found that aquatic HIIT reduced sympathetic nerve activity and catecholamine release due to the influence of water temperature, thus reducing blood pressure (Rodriguez et al., 2011). Therefore, the effect of aquatic HIIT is better under the same conditions. Different types or intensities of exercise interventions are associated with different anterograde and retrograde shear stress patterns (Green, 2009). The intensity of HIIT during high-intensity exercise enhances the blood supply capacity of the heart, accelerates the blood flow speed, enhances the stimulation of the blood vessel wall, and the vascular smooth muscle passively contracts and diastole rapidly under mechanical stress to meet the needs of blood flow (Ramos et al., 2015). Thus, long-term exercise led to better improvements in WSS and PSV of middle-aged men. In addition, WSS can also stimulate endothelial cells to produce vascular regulatory factors and continuously improve the vascular microenvironment, so that cardiovascular function can be well improved (Phillips et al., 2015). This study found that aquatic HIIT accelerated blood flow and enhanced stimulation of blood vessel wall due to the influence of water flow and water temperature on body surface blood vessels. Therefore, aquatic HIIT can improve WSS and PSV better than land HIIT.

It has been reported that regular exercise can enhance vascular endothelial function in men by reducing oxidative stress and maintaining nitric oxide bioavailability (Seals et al., 2019). The improvement mechanism of HIIT exercise on vascular endothelial function mainly includes the increase of blood flow and shear stress, and the decrease of reactive oxygen species production, thus increasing the bioavailability of nitric oxide in vascular endothelium, maintaining vascular homeostasis, delaying the rate of vascular degeneration, and ultimately affecting endothelial dysfunction (DeSouza et al., 2000). This study suggests that aquatic HIIT produces higher shear stress and a greater response to inducing nitric oxide production than land HIIT. Therefore, aquatic HIIT can effectively induce vascular endothelial factors, thus making the improvement of vascular endothelial function more obviously. Studies have shown that regular exercise can improve the arterial hardness of middle-aged and elderly people under different health conditions, delay the development of atherosclerotic plaque, and reduce the risk of cardiovascular disease (Francis, 1996). The improvement of arterial hardness by exercise can stimulate the growth, differentiation and synthesis of vascular endothelial cells and smooth muscle cells through blood flow stimulation, and promote the thickening of blood vessel wall and the remodeling of blood vessel structure (Green and Smith, 2018). Therefore, regular exercise can not only promote the remodeling of artery structure, but also protect vascular endothelial cells and reduce vascular endothelial damage by improving mitochondrial function and inhibiting inflammatory response, so as to prevent and treat atherosclerosis and related cardiovascular diseases. This study found that there was no significant difference in PWV between the groups of aquatic and land HIIT after 8 weeks, which may be related to the duration of exercise intervention. The physical characteristics of the water environment at 8 weeks were not enough to cause significant improvement in PWV. Whether the difference between the groups of aquatic and land HIIT can be caused by lengthening the exercise intervention period remains to be further explored. Therefore, the PWV level of middle-aged men could have effectively improved in both the aquatic and the land HIIT groups at 8 weeks, however, there was no significant difference between the improvement of the aquatic and the land groups.

The sample consisted of homogenous middle-aged men, which may limit its general applicability to other populations. Further studies could include more participants to support our hypothesis.

## 5 Conclusion

8 weeks of aquatic and land HIIT had different benefits in improving body composition, hemodynamics and vascular function in middle-aged men. In particular, aquatic HIIT was better than land HIIT in improving body fat percentage, hemodynamics and vascular endothelial function in middle-aged men due to the effects of water pressure and water temperature. Therefore, aquatic HIIT can be a feasible and effective method to improve cardiovascular functions better.

## Data Availability

The original contributions presented in the study are included in the article/Supplementary material, further inquiries can be directed to the corresponding author.
